# The impact of area residential property values on self-rated health: A cross-sectional comparative study of Seattle and Paris

**DOI:** 10.1016/j.pmedr.2016.05.008

**Published:** 2016-05-17

**Authors:** Junfeng Jiao, Adam Drewnowski, Anne Vernez Moudon, Anju Aggarwal, Jean-Michel Oppert, Helene Charreire, Basile Chaix

**Affiliations:** a School of Architecture, University of Texas at Austin, Austin, TX, United States; b Center for Public Health Nutrition, University of Washington, Seattle, WA, United States; c Urban Form Lab, College of Built Environments, University of Washington, Seattle, WA, United States; d the Nutrition Epidemiology Unit, Paris 13 University, Paris, France; e The Institute of Urbanism of Paris, Paris 12 Val de Marne University, Paris, France; f Pierre Louis Institute of Efpidemiology and Public Health, Paris, France

**Keywords:** Socioeconomic status, Health disparities, Adult population, BMI

## Abstract

This study analyzed the impact of area residential property values, an objective measure of socioeconomic status (SES), on self-rated health (SRH) in Seattle, Washington and Paris, France. This study brings forth a valuable comparison of SRH between cities that have contrasting urban forms, population compositions, residential segregation, food systems and transportation modes. The SOS (Seattle Obesity Study) was based on a representative sample of 1394 adult residents of Seattle and King County in the United States. The RECORD Study (Residential Environment and Coronary Heart Disease) was based on 7131 adult residents of Paris and its suburbs in France. Socio-demographics, SRH and body weights were obtained from telephone surveys (SOS) and in-person interviews (RECORD). All home addresses were geocoded using ArcGIS 9.3.1 (ESRI, Redlands, CA). Residential property values were obtained from tax records (Seattle) and from real estate sales (Paris). Binary logistic regression models were used to test the associations among demographic and SES variables and SRH. Higher area property values significantly associated with better SRH, adjusting for age, gender, individual education, incomes, and BMI. The associations were significant for both cities. A one-unit increase in body mass index (BMI) was more detrimental to SRH in Seattle than in Paris. In both cities, higher area residential property values were related to a significantly lower obesity risk and better SRH. Ranked residential property values can be useful for health and weight studies, including those involving social inequalities and cross-country comparisons.

## Highlights

• We studied the impact of area property values on health in Seattle and Paris.
• Higher area property values associated with better SRH in both cities
• Ranked area property values can be useful for health and weight studies.
• BMI was more detrimental to SRH in Seattle than in Paris.

## 1 Introduction

Social and economic variables can exert a powerful influence on body weight and health (Adler et al., 1993, Mustard et al., 1997, Lantz et al., 1998, Frumkin, 2002, Rundle et al., 2009). In general, higher socioeconomic status (SES) is associated with better health and lower body weights (Pampel et al., 2010, Williams et al., 2013). Using three self-reported measures of SES (occupation, education, and income), a recent study of 29 countries in the European Union (EU) showed that socioeconomic inequalities did affect self-rated health (SRH), with education having the strongest impact (Alvarez-Galvez et al., 2013). People living in Scandinavian and Anglo-Saxon countries appeared to be less affected by SES variables as compared to those living in Eastern and Southern Europe (Alvarez-Galvez et al., 2013).

This paper makes three distinct contributions. First, disparities in SRH can also be observed at the microscale. While it is instructive to compare social disparities across countries, it may also be useful to compare SRH across cities that differ in urban form, population composition, residential segregation, food systems and transportation modes. The question is how the socioeconomic determinants of health affect SRH across cities and their neighborhoods. The present analyses compare Paris and Seattle.

Second, past educational attainment and current income may not capture the multiple facets of SES (Lee, 2004, Pollack et al., 2013). Given that many respondents fail to provide data on incomes, some studies (Delpierre et al., 2012) used education only. Hanibuchi et al. included “class identification” as a unique, fourth variable in their analysis of the effects of SES on SRH in four East Asian countries (Hanibuchi et al., 2012). In this study, we have introduced residential property values, obtained from tax records, as a novel measure of SES for use in health studies (Moudon et al., 2011). Residential property values can complement composite scores of area deprivation, based on multiple variables, usually at the census tract level (Black et al., 1988, Morris and Carstairs, 1991, Singh-Manoux et al., 2006, Pampalon et al., 2009). Residential property values are a reliable index of neighborhood assets and a reflection of individual SES (Moudon et al., 2011, Rehm et al., 2012).

Third, this research adds to the growing literature of studies examining the interaction between urban-form-related SES variables, functional limitations and SRH. Obesity rates, determined by body mass indexes, are linked to social inequalities and obesity prevalence is higher in the US than in France (Drewnowski et al., 2013), with 32% of all Americans above 20 years of age being classified as obese (Rundle et al., 2009).

## 2 Methods and procedures

### 2.1 Population samples in SOS and RECORD studies

The Seattle Obesity Study (SOS), conducted in 2008–2009, was a population-based survey of 2001 adult residents of King County, WA (Aggarwal et al., 2012). Detailed methodology of this study has been published (Moudon et al., 2011, Aggarwal et al., 2012). A stratified random sampling scheme ensured adequate representation by income range and race/ethnicity. Randomly generated telephone numbers were matched with residential addresses using commercial databases. A pre-notification letter was mailed out and telephone calls were placed in the afternoons and evenings by trained, computer-assisted interviewers. An adult member of the household was randomly selected to be the survey respondent. Exclusion criteria were age < 18 years, cell phone numbers, and mismatch in address data from the vendor and from self-report. The study protocol was approved by the University of Washington (UW) Institutional Review Board. Analyses were based on 1394 respondents for whom complete data (including incomes) were available.

The first wave of the RECORD Study (www.record-study.org), conducted in 2007–2008, was based on 7290 eligible persons attending 2-hour preventive checkups conducted by the Centre d'Investigations Préventives et Cliniques in four of its health centers located in Paris, Argenteuil, Trappes, and Mantes-la-Jolie (Chaix et al., 2012). Participants were affiliated with the French National Health Insurance System for Salaried Workers, which offers a free medical examination every 5 years to all working and retired employees and their families. People who take part in preventive health checkups do so following the referral of their family or workplace physician, or on the advice of peers. Eligibility criteria were as follows: age 30–79 years, ability to complete study questionnaires, and residence in one of the 10 (out of 20) pre-selected administrative districts of Paris or in 111 other municipalities of the metropolitan area. The intent was to oversample disadvantaged municipalities and have sufficient power to compare urban and suburban areas. Among eligible respondents, based on age and residence, 10.9% were not selected because of linguistic or cognitive difficulties. From those who were eligible, 83.6% agreed to participate and completed data collection protocol for a total of 7131 respondents.

**Fig. 1:**
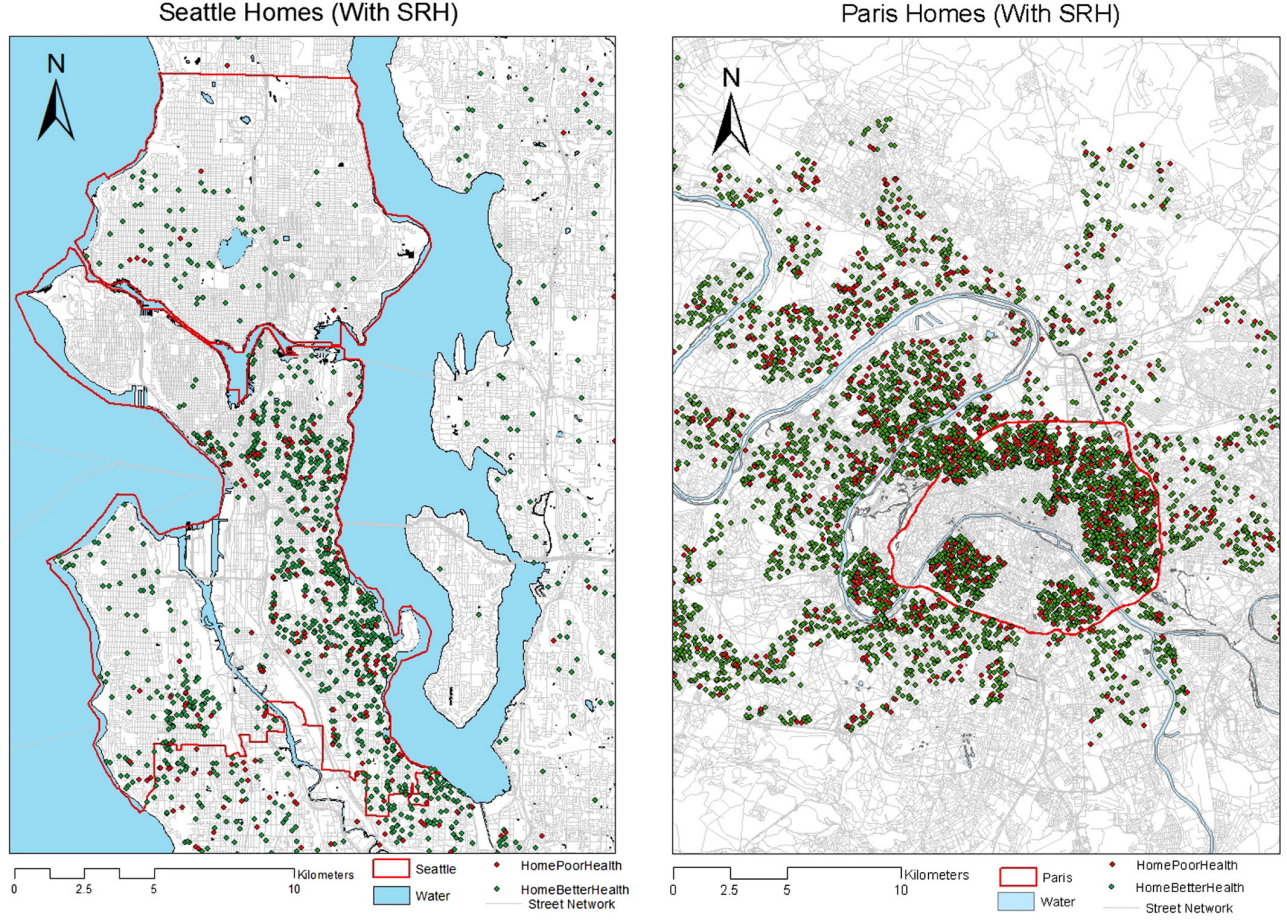
Respondents' home locations in Seattle and Paris, red (within the city limit), black (outside of the city limit). (For interpretation of the references to color in this figure legend, the reader is referred to the web version of this article.)

The SOS recruited participants from the city of Seattle and the urban growth boundary of King County. In the RECORD study, participants came from the city of Paris intramuros and the suburb. The spatial distributions of these samples in Seattle and Paris can be found in Fig. 1.

### 2.2 Demographic, socio-economic, weight and health measures

Both the SOS and the RECORD Study collected data on age, gender, education, income, and household size. In both studies, self-reported individual SES was based on education and income. Education was organized into 3 comparable categories for analytical purposes: high school or less, some college, and college graduates or higher (effectively > 12 y, 12–16 y and > 16 y). Annual household income was divided into tertiles based on the most recent census information in the two cities and the underlying distribution of the data. Questions about marital status and household size were comparable in the SOS and the RECORD Study. The dichotomous variable “living alone or not” was based on reported household size in the SOS and on a question regarding cohabitation status in the RECORD Study.

Participant heights and body weights were obtained through telephone self-report in the SOS and measured by a nurse in the RECORD Study using a wall-mounted stadiometer and calibrated scales (Thomas et al., 2005). Body mass index was calculated as body weight (in kilograms) divided by the square of height (in meters). The cut-points for overweight (BMI > 25–30) and obesity (BMI > 30) were the same in both studies. BMI was also analyzed as a continuous variable. Measures of perceived health status were based on a fully-anchored 5-point category scale in the SOS and on a semi-anchored 11-point category scale ranging from 0 to 10 in the RECORD Study. These two category scales are comparable in psychometric terms. Detailed survey and variable selection methods can be found in the published journals (Moudon et al., 2011, Aggarwal et al., 2012, Chaix et al., 2012).

### 2.3 Geocoding of home addresses

Procedures for geocoding of home addresses were specific to each country but each resulted in spatial coordinates that were used to calculate network distances. In the SOS, home addresses were geocoded to the centroid of the home parcel using the 2008 King County Assessor parcel data, by means of standard methods in ArcGIS 9.3.1 (ESRI, Redlands, CA). Addresses that failed the automatic geocoding (60% of the samples were geocoded based on 100% match score) were manually matched using a digital map environment, augmented by online resources such as GoogleMaps, QuestDEX and Yelp. Each home point was checked for plausibility and accuracy. In the RECORD Study, home addresses in 2007–2008 were geocoded using the Geoloc software of the National Institute of Statistics and Economic Studies. Research assistants rectified all incorrect or incomplete addresses with the participants by telephone. Additional data were obtained from local authorities. Spatial coordinates and block group codes were identified for 100% of the sample.

### 2.4 Residential property values

Property values were handled differently in the SOS and in the RECORD Study. The residential property values for Seattle were based on county tax assessor data for individual parcels, whereas the data for Paris were based on prices of properties sold over a given period of time.

In the SOS, assessed residential property values were obtained from the 2008 King County tax assessor parcel database. Property values are determined by the combined value of both land and improvements (buildings and other structures), and based on recent local sales data. For home parcels with multiple residential units (e.g., apartment buildings), assessed value per unit was calculated as the sum of a parcel's land and improvement values divided by the number of residential units on the parcel. Tax assessment in Washington State aims to estimate the full market value of a given property (King County Department of Assessments). The primary variable of interest was the mean assessed property value per residential unit in the 833 m radius buffer around the each respondent's home.

In the RECORD Study, residential property values were assessed on the basis of notary data obtained from Paris-Notaries related to the price at which properties (including land, and improvements) were sold. Mean value of dwellings sold within a French IRIS (similar size to a US block) between 2003 and 2007 was computed (after accounting for variations of price levels between years) and captured with a 500 m radius buffer around each respondent's home. The mean score was categorized by quartiles.

### 2.5 Statistical analyses

Separate binary logistic regressions were conducted to examine the impact of demographic, socioeconomic, average area property value, and BMI on SRH. In detail, Model 1 only included demographic and socioeconomic variables. In Model 2, average area property value was added. Model 3 included all the variables in Model 2 and added three categories of BMI (normal, overweight and obese). An alternative model also considered BMI as a continuous variable (results not shown). All analyses were conducted using Stata 10.0.

## 3 Results

### 3.1 Participants' characteristics: SOS and RECORD

Table 1, Table 2 show that the SOS sample was predominantly female (61%), married (67%), college educated (57%), and > 45 years old (75%). Annual household income was ≥$50 K for 60% of the sample (median for King County was $53,157 based on 2000 Census data). With an average BMI of 26.60, the obesity rate of the participants was 21%, compared to the county-wide estimate of 21.5% for King County in the 2009 BRFSS, and “poor or fair” SRH was reported by 12% of the SOS sample.

RECORD Study participants were predominantly male (65%), married (70%), and > 45 years old (65%). Only two out of five persons (38%) were college educated. With an average BMI of 25.47, the obesity rate was 12%, which was equal to the national estimate for France (Charles et al., 2008, Castetbon et al., 2009, Diouf et al., 2010). “Poor or fair” health status was reported by 15% of the RECORD sample, which was an increase of 3% compared to the SOS sample.

Living in the city of Seattle, as opposed to the suburbs, was associated with higher education, living alone (39% vs. 27%) and with lower obesity rates (18% vs. 24%). Living in the city of Paris, as opposed to the suburbs, was associated with higher education, living alone (37% vs. 27%), and with lower obesity rates (9% vs. 14%).

### 3.2 SRH and sociodemographics: SOS and RECORD

Binary logistic regression analysis examined the associations between SRH, demographic and SES variables, including residential property values (Table 3). Model 1 showed that education and incomes were positively correlated with SRH in both cities. However, being a male was negatively correlated with SRH in Seattle but positively correlated with SRH in Paris. Models 2 and 3 showed that SRH was linked positively and independently to all three SES variables: education, incomes, and area residential property values. In both the SOS and the RECORD Study, higher education and incomes were each independently associated with higher odds of reporting good or excellent health. Living in an affluent neighborhood was also associated with higher odds of reporting good or excellent health. For these three individual/neighborhood socioeconomic variables, in Seattle as in Paris, the associations were systematically dose–response, even after mutual adjustment. Being a male was not significantly correlated with SRH in Seattle but still positively related with SRH in Paris.

The impact of SES factors on SRH was greater in Seattle than in Paris for the three SES variables, even after mutual adjustment. In Seattle, residents living in the top quartile of property values were 59% less likely to report a fair/poor health status compared to residents living the bottom quartile of property values. In Paris, the corresponding reduction was only 23% (Model 3). In Seattle, individuals having a college degree or higher were 59% less likely to report a poor health status compared to samples with high school or less education. In Paris, the corresponding reduction was 42%. Similarly, the effect of household income was also stronger in Seattle than in Paris.

Living within the city limits was not related to SRH in either of the cities. There was some evidence that the relationship between gender and SRH was in the opposite direction in Seattle and in Paris. While men tended to more often have a fair/poor vs. good/excellent SRH in Seattle, they less often reported a fair/poor SRH than women in Paris.

Being obese was negatively correlated with SRH; however, the detrimental obesity effect was much greater in Seattle than in Paris (Model 3). Being overweight was negatively correlated with SRH in Paris but was not related to SRH in Seattle. Alternative models also tested the impacts of BMI as a continuous variable on SRH. The results showed that a one unit BMI increase in Seattle would increase the likelihood of reporting a poor/fair health status by 9% (95% CI: 6%–12%). In Paris, the corresponding increase was only of 5% (95% CI: 3%–7%). We also added a quadratic term for BMI, but the 95% CI of the coefficient overlapped 0 in both cities, and this quadratic term was dropped from the final model reported in Table 3, Model 3.

## 4 Discussion

The SOS and the RECORD Study are among the first studies to examine interactions between SES variables, SRH, and obesity rates. In both Seattle and Paris, SRH was strongly linked to SES variables, both reported and objective. Remarkably, regression models provided very comparable findings for both cities, suggesting that the social mechanisms underlying SRH may be the same. In both cities, low area property values, low educational attainment and low incomes had significant impacts on SRH.

This comparative study also showed that the impact of socioeconomic disparities on SRH was larger in Seattle than in Paris (income, education and residential property value showed stronger impacts on SRH than those in Paris). Significantly, obesity was both higher and also appeared to be more detrimental to SRH in Seattle than in Paris. In Europe, obesity among women was shown to be more strongly and more consistently related to education, rather than to occupation or incomes (Mackenbach et al., 2008, Roskam and Kunst, 2008). In the SOS, obesity among women was most strongly associated with education and low residential property values (Drewnowski et al., 2014).

The present approach to measuring SES was based on property values within an area or neighborhood buffer zone around each respondent's residential address, which required reconciling different methodologies. In the US, property values are available from tax rolls that are in the public domain. In Seattle, property values are derived from recent sales because by law, property assessment must equate sales value. In France, comparable data were obtained from tax files and notaries, based on recent sales. Property values, whether from tax rolls or real estate sales, can complement self-reports of education and incomes in the US (Braveman, 2006) and the additional measure of occupation used in the EU (Heritage, 2009). One common problem with large-scale health surveys is that income data are inaccurate or missing. In contrast, property values are commonly available at a small spatial resolution.

The use of the novel methodology requires some new distinctions of what constitutes individual SES as opposed to area SES. There has been some debate as to whether SES derived from administrative unit areas was more or less important than SES derived from individual neighborhoods (Steenland et al., 2004). Pampalon et al. found that associations between SES and health varied depending on the size of the administrative unit from which SES measures were obtained (Pampalon et al., 2009). Researchers have tried to circumvent these issues by spatially buffering census units around the individual's home and allocating the values of each census unit proportionally to their area in the buffer (Lovasi et al., 2008). Others have adjusted the buffer to take into consideration the size of the units' population (Chaix et al., 2005). Others weighed census units based on the number of respondents living in them (Tatalovich et al., 2006). Although participant-level home-based buffered measures can take into consideration or adjust to different “neighborhood of influence” near the individual, and thereby allocate more realistic “neighborhood” values to that individual, they still draw from data that have been aggregated within the boundaries of their original spatial unit. The bias that spatial aggregation introduces to any type of measurement should not be overlooked in research (Lancaster, 2006, Wakefield and Shaddick, 2006). Previous work based on the RECORD Study has used socioeconomic census data or income data from tax registries geocoded at the building level (street address level) to assess area socioeconomic status in specifically designed buffers of different sizes (Heritage, 2009, Chaix et al., 2012, Leal et al., 2012, Karusisi et al., 2013). Clearly, using less aggregated property value helped researchers better capture an individual's exposures to the surrounding neighborhood environments (Chaix et al., 2009, Kwan, 2012). Finally, Moudon and colleagues found that compared to administrative unit-level SES measures, individual neighborhood built environment (especially property value) was a better predictor for residents' SRH (Moudon et al., 2011).

With respect to neighborhood influences on health, Cummins and colleagues found that in the UK, fair to very bad SRH was significantly associated with poor neighborhood-level physical residential environment and lower access to private transport (Cummins et al., 2005). Furthermore, low income neighborhoods have additional characteristics such as greater numbers of liquor stores, fewer options for healthy foods, and a greater risk of being targeted by tobacco companies (Pampel et al., 2010). Also in the UK, Dunstan and colleagues found that after adjusting for both the individual characteristics and area deprivation, respondents in the areas of the poorest neighborhood quality were more likely to report poor SRH compared to those living in areas of the highest quality (Dunstan et al., 2013). Bonnefoy found that neighborhood housing conditions, especially noise annoyance, had a direct impact on their residents' SRH (Bonnefoy, 2007). Yen et al. also determined that neighborhood built environment (traffic, noise, trash, smells, and smoke) had a direct impact on people's SRH (Yen et al., 2006). Further, residents living in high-income neighborhoods did report a better physical quality of life. Clearly more research needed to reveal the complicated relationships between neighborhood built environment and SRH.

The links between obesity and SRH require some discussion. Delpierre et al. have reported that functional limitations in the US were associated more strongly with poor SRH in the most educated men than in the least educated, as compared to France (Delpierre et al., 2012). This finding is in contrast with one previous comparative study between Seattle and Paris that focused on body mass index, which did not report systematically stronger socioeconomic contrasts in body weight in the US than in France (Drewnowski et al., 2013). Future research should investigate the extent to which the larger socioeconomic differences in SRH in Seattle, in comparison to Paris, are attributable to objective health conditions and/or to perceptions and interpretation of one's health status. It would also be noteworthy to further assess whether such greater differences in the US (as compared to France) are driven by the relatively more favorable health situation of affluent people or by the worse health status of the poorest in the US.

The relation between obesity and SRH was found to be markedly stronger in Seattle than in Paris where the population is leaner. A hypothesis was that it may be due to the much higher percentage of obese population in Seattle than in Paris (21% vs. 12%). A separate model coding BMI as a continuous variable showed that a one unit increase in BMI was associated with a larger deterioration of SRH in Seattle than in Paris. Thus, this finding further suggests that there may also be differences between the two cities in the perception of how a high body mass index is detrimental to health.

Both studies had limitations. The SOS sample, based on a random landline-telephone survey (standard BRFSS procedure) was older, better educated and had a higher proportion of females than the background population. Heights and body weights were based on self-reported data, which may have resulted in some discrepancies due to underreporting body weights. A recent publication focused on a comparable Seattle-based cohort also showed a good correspondence between self-reported and measured heights and weights (Tang et al., 2016).

The Seattle sample might exclude some low SES subjects who do not have landline telephones. Conversely, the Paris sample was employment-based, with an over-representation of males, and people with higher education attainment and coming from more affluent and lower-density neighborhoods. Furthermore, because of limitations in data availability, the authors could not include the same built environment or spatial variables in both models.

Both studies were cross-sectional, limiting our ability to draw causal inference from the observed associations. Nonetheless, the present Seattle–Paris comparisons are valuable given the different socioeconomic, behavioral and environmental context of the two cities. The relative influences of income, education, and property values on SRH can provide additional insight into the contextual socioeconomic determinants of SRH.

## Conflict of interest statement

The authors declare that there are no conflicts of interest.

## Figures and Tables

**Table 1:** Comparison of key variables between Seattle and Paris.

	Seattle Obesity Study SOS	Paris RECORD
Age	Age groups	Age groups
	< 45 y	< 45 y
45 to < 65 y	45 to < 65 y
≥ 65 y	≥ 65 y
Gender	Male/female	Male/female
Living alone	Yes/no	Yes/no
Household income	Annual household income ($/y)	Monthly household income (€/mo)
	Tertile 1 (<$50,000)	Tertile 1 (<€1200)
Tertile 2 (≥$50,000–<$100,000)	Tertile 2 (≥€1200–<€2200)
Tertile 3 (≥$100,000)	Tertile 3 (≥€2200)
Education		
	High school or less	Primary school and lower secondary school or less
Some college	Higher secondary school and lower tertiary school
College graduates or higher	Higher tertiary school BAC + 2
Body weight	BMI	BMI
	Overweight (BMI ≥ 25 &≤ 29.9)	Overweight (BMI ≥ 25 & ≤ 29.9)
Obese (BMI ≤ 30 kg/m^2^)	Obese (BMI ≥ 30 kg/m^2^)
Non obese or overweight (BMI < 25 kg/m^2^)	Non obese or overweight (BMI < 26 kg/m^2^)
Self rated health		
Measurements	Fair/poor	Fair/poor: Scale 0–5
Good/ very good/ excellent	Good/ very good/ excellent: Scale 6–10
Residential property values	Assessed property values 833 m circular buffer.	Residential sales data 500 m circular buffer, measured on a 1–1000 scale.
	$70,381–193,106	1–301
$193,107–248,011	302–420
$248,012–334,445	421–536
$334,446–1,086,587	537–1000
Location	City/suburbs	City/suburbs
	Seattle (area: 218 square km, population: 652,405, density: 3000 persons/square km)	Paris (area: 106 square km, population: 2,244,000, density: 21,132 persons/square km)
	Suburbs (area: 14,990 square km, population: 3,020,000, density: 202 persons/square km)	Suburbs (area: 17,068 square km, population: 10,097,418, density: 591 persons/square km)

**Table 2:** Distribution of study participants by demographic and socioeconomic variables in the SOS and RECORD studies.

	Seattle Obesity Study (SOS)			RECORD study		
Seattle total N = 1394	Seattle city N = 707	Seattle suburb N = 687	Paris total N = 7131	Paris city N = 2044	Paris suburb N = 5087
*Gender*						
Men	542 (39%)	265 (38%)	277 (40%)	4658 (65%)	1315 (64%)	3343 (66%)
Women	852 (61%)	442 (62%)	410 (60%)	2473 (35%)	729 (36%)	1744 (34%)

*Age strata (years)*						
18–<45	356 (25%)	203 (29%)	153 (22%)	2535 (35%)	746 (37%)	1789 (35%)
45–<65	721 (52%)	359 (51%)	362 (53%)	3762 (53%)	1022 (50%)	2740 (54%)
≥ 65	317 (23%)	145 (20%)	172 (25%)	834 (12%)	276 (14%)	558 (11%)

*Living alone/not*						
Alone	463 (33%)	275 (39%)	188 (27%)	2136 (30%)	751 (37%)	1385 (27%)
With others	931 (67%)	432 (61%)	499 (73%)	4995 (70%)	1293 (63%)	3702 (73%)

*Household income*						
Tertile 1	567 (41%)	298 (42%)	269 (39%)	2706 (38%)	562 (28%)	2153 (43%)
Tertile 2	468 (34%)	228 (32%)	240 (35%)	2451 (35%)	770 (38%)	1698 (33%)
Tertile 3	359 (25%)	181 (26%)	178 (26%)	1936 (27%)	712 (35%)	1207 (24%)

*Education*						
High school or less	255 (18%)	94 (13%)	161 (23%)	2303 (36%)	480 (24%)	1823 (36%)
Some college	351 (25%)	157(22%)	194 (28%)	2098 (30%)	560 (28%)	1538 (31%)
College graduates or higher	788 (57%)	456 (66%)	332 (49%)	2672 (38%)	990 (48%)	1682 (33%)

*Residential property value*						
Quartile 1	348 (25%)	100 (14%)	248 (36%)	1771 (25%)	352 (17%)	1419 (28%)
Quartile 2	331 (24%)	199 (28%)	132 (19%)	1766 (25%)	747 (37%)	1019 (20%)
Quartile 3	355 (26%)	200 (28%)	155(23%)	1771 (25%)	533 (26%)	1238 (25%)
Quartile 4	360 (25%)	208 (30%)	152 (22%)	1775 (25%)	390 (19%)	1385 (27%)

*BMI*						
Overweight (25 ≤ BMI < 29.9)	462(33%)	221 (31%)	241(35%)	2673(37%)	724 (36%)	1949 (39%)
Obese (BMI ≥ 30 kg/m^2^)	295 (21%)	128 (18%)	167 (24%)	880 (12%)	192 (9%)	688 (14%)
Non obese or overweight (BMI < 25 kg/m^2^)	637 (46%)	358 (51%)	279 (41%)	3678 (50%)	1128 (55%)	2450 (43%)

*Perceived health status*						
Fair/poor	171 (12%)	83 (12%)	88 (13%)	1086 (15%)	257 (13%)	829 (16%)
Good/very good/excellent	1223 (88%)	624 (88%)	599 (87%)	6045 (85%)	1787 (87%)	4258 (84%)

**Table 3:** Binary logistic regression with robust error variance for SRH (fair/good) with individual and area SES variables.

Independent variables	Seattle Obesity Study (SOS) (N = 1394)						RECORD Study (N = 7131)					
Model 1		Model 2		Model 3		Model 1		Model 2		Model 3	
RR	95% CI	RR	95% CI	RR	95% CI	RR	95% CI	RR	95% CI	RR	95% CI
*Household income*												
Tertile 1	Ref		Ref		Ref		Ref		Ref		Ref	
Tertile 2	**0.47**	**0.31, 0.71**	**0.49**	**0.32, 0.74**	**0.51**	**0.33, 0.78**	**0.50**	**0.42, 0.58**	**0.50**	**0.42, 0.58**	**0.51**	**0.43, 0.60**
Tertile 3	**0.27**	**0.15, 0.50**	**0.33**	**0.18, 0.61**	**0.37**	**0.20, 0.68**	**0.39**	**0.32, 0.48**	**0.39**	**0.32, 0.48**	**0.40**	**0.33, 0.49**

*Education completed*												
High school or less	Ref		Ref		Ref		Ref		Ref		Ref	
Some college	**0.61**	**0.39, 0.93**	**0.62**	**0.40, 0.95**	0.66	0.42, 1.04	**0.64**	**0.54, 0.75**	**0.66**	**0.56, 0.77**	**0.67**	**0.57, 0.79**
College graduate or higher	**0.35**	**0.23, 0.55**	**0.38**	**0.24, 0.59**	**0.41**	**0.26, 0.64**	**0.53**	**0.45, 0.64**	**0.56**	**0.46, 0.67**	**0.58**	**0.49, 0.70**

*City or suburbs*												
Suburbs	Ref		Ref		Ref		Ref		Ref		Ref	
City	0.95	0.67, 1.35	1.07	0.74, 1.56	1.15	0.78, 1.69	0.89	0.76, 1.04	0.89	0.76, 1.05	0.92	0.78, 1.08

*Gender*												
Female	Ref		Ref		Ref		Ref		Ref		**Ref**	
Male	**1.42**	**1.01, 2.01**	1.39	0.98, 1.96	1.35	0.94, 1.93	**0.63**	**0.55, 0.73**	**0.63**	**0.54, 0.72**	**0.61**	**0.53, 0.70**

*Area property values*												
Quartile 1			Ref		Ref				Ref		Ref	
Quartile 2			0.81	0.51, 1.28	0.82	0.51, 1.30			0.90	0.75, 1.08	0.94	0.81, 1.08
Quartile 3			0.84	0.53, 1.34	0.89	0.55, 1.43			0.83	0.69, 1.00	**0.87**	**0.75, 0.99**
Quartile 4			**0.37**	**0.19, 0.70**	**0.41**	**0.21, 0.79**			**0.78**	**0.63, 0.95**	**0.77**	**0.66, 0.89**

*BMI (categorical)*												
Normal					Ref						**Ref**	
Overweight					1.06	0.67, 1.67					**1.32**	**1.13, 1.54**
Obese					**3.39**	**2.23, 5.15**					**1.89**	**1.55, 2.29**

Model 1: Adjusted for age, gender, living alone or not, living within city limits or not, income, and education,

Model 2: Model 1 + average area property value.

Model 3: Model 2 + BMI (categorical), separated models also tested the impact of BMI as a continuous variable on SRH (results not shown). Significance at the 0.05 level.
